# Extra-Articular Manifestations in Patients with Ankylosing Spondylitis: Baseline Characteristics from the RBSMR Study

**DOI:** 10.31138/mjr.33.3.316

**Published:** 2022-09-30

**Authors:** Najlae El Ouardi, Julien H. Djossou, Laila Taoubane, Mohamed A. Ghassem, Hamza Toufik, Abderrahim Majjad, Siham Sadni, Ihsane Hmamouchi, Redouane Abouqal, Rachid Bahiri, Fadoua Allali, Imane El Bouchti, Imad Ghozlani, Hasna Hassikou, Taoufiq Harzy, Linda Ichchou, Ouafa Mkinsi, Redouane Niamane, Abdellah El Maghraoui, Lahsen Achemlal

**Affiliations:** 1 Department of Rheumatology, Military Hospital Mohammed V, Ibn Sina University Hospital, Rabat, Morocco,; 2 Laboratory of Biostatistical, Clinical and Epidemiological Research, Faculty of Medicine and Pharmacy, Mohammed V University, Rabat, Morocco,; 3 Department of Rheumatology, Provincial Hospital of Temara, Morocco,; 4 Department of Rheumatology A, El Ayachi Hospital, Ibn Sina University Hospital, Salé, Morocco,; 5 Department of Rheumatology B, El Ayachi Hospital, Ibn Sina University Hospital, Salé, Morocco,; 6 Department of Rheumatology, Arrazi University Hospital, Marrakech, Morocco,; 7 Department of Rheumatology, University Hospital of Agadir, Morocco,; 8 Department of Rheumatology, Military Hospital Moulay Ismail, Hassan II University Hospital, Meknès, Morocco,; 9 Department of Rheumatology, Hassan II University Hospital, Fès, Morocco,; 10 Department of Rheumatology, Mohammed VI University Hospital, Oujda, Morocco; 11 Department of Rheumatology, Ibn Rochd University Hospital, Casablanca, Morocco,; 12 Department of Rheumatology, Military Hospital Avicenne, Mohammed VI University Hospital, Marrakech, Morocco,; 13 Private Medical Office, Rabat, Morocco

**Keywords:** ankylosing spondylitis, extra-articular manifestations, acute anterior uveitis, psoriasis, inflammatory bowel disease

## Abstract

**Objectives:**

To describe and analyse the prevalence of extra-articular manifestations (EAMs) including acute anterior uveitis (AAU), psoriasis and inflammatory bowel disease (IBD) in patients with ankylosing spondylitis (AS) in the Moroccan registry of biological therapies in rheumatic diseases RBSMR (Registre des Biothérapies de la Société Marocaine de Rhumatologie).

**Methods:**

A cross-sectional, multicentre and analytical study based on the RBSMR database, which included 170 AS. Incidence rates for the development of AAU, psoriasis and IBD were calculated, and risk factors were analysed.

**Results:**

Prevalence of EAMs in AS was 13.5%, 4.7% and 11.2% for AAU, psoriasis and IBD respectively. No significant differences were found while establishing a comparison of the prevalence of these EAMs between AS patients with and without peripheral arthritis. Interestingly, AAU was the most common EAM, and was positively associated in multivariable regression with family history of spondyloarthritis (OR= 7.21, CI 95%: 2.23–23.24).

**Conclusions:**

AAU was the leading EAM in patients with AS included in the Moroccan biotherapy registry (RBSMR) and it was associated with family history of spondyloarthritis.

## INTRODUCTION

Ankylosing Spondylitis (AS) is a chronic inflammatory rheumatism primarily affecting the axial skeleton. Peripheral joint involvement occurs in 22.9% of the patients.^1^ AS is also termed radiographic axial spondyloarthritis according to the new classification criteria developed by Assessment in Spondyloarthritis International Society (ASAS).^2,3^ AS occurs in 0.2–1.2% of the general population and it is 2.5-times more common in men than women.^4^ The disease course is usually accompanied by extra-articular manifestations (EAMs) comprising acute anterior uveitis (AAU), psoriasis and inflammatory bowel disease (IBD).^5^ In the last decades, the attention to these EAMs in AS had increased. First, the presence of AAU, psoriasis and IBD were included in the ASAS diagnostic criteria.^6^ In addition, the prognosis of AS and health outcomes including quality of life, work outcomes and resource usage could be affected by the presence of one or more EAMs. Moreover, EAMs in patients with AS may influence the choice of treatment.^7^ Furthermore, previous studies have focused on the characteristics of EAMs and have demonstrated that their prevalence vary substantially, whereas, variation cannot be explained since associated risk factors are still not well clarified.^7^ Therefore, the objectives of this study were to describe the prevalence of the EAMs (AAU, psoriasis and IBD) in patients with AS at the time of recruitment into the RBSMR study and to determine the characteristics associated with their development.

## METHODS

### RBSMR record

The RBSMR (Registre des Biothérapies de la Société Marocaine de Rhumatologie) is a registry of biological therapies in rheumatic diseases of the Moroccan Society of Rheumatology. It is a historical, prospective, and multicentre registry, which includes 10 departments of rheumatology in university medical centres. The patients recruited in the registry, had an age of over 18 years. They were diagnosed with Rheumatoid Arthritis (RA) or Spondyloarthritis (SpA) and treated by biotherapy (initiation or ongoing biotherapy) in different Moroccan university medical centers. The inclusion period was from May 2017 to January 2019 and the scheduled follow-up is 3 years. The total number of patients included in the registry was 440 patients; 419 among them were validated (225 RA/194 SpA). The primary objective of the RBSMR record was to assess the safety of biologics used in RA or SpA management. The secondary objectives were to evaluate the effectiveness of biologics in the real-world and to evaluate the impact of biologics on the patient’s quality of life.

### Study

We performed a cross-sectional, multicentre, and analytical study using the RBSMR database, which included 170 patients AS who fulfilled the axial ASAS criteria with radiographic evidence of sacroiliitis. The study was designed to describe the prevalence of EAMs included in the ASAS diagnostic criteria (psoriasis, AAU and IBD) of all AS included in the RBSMR registry at the time of inclusion into the study and to analyse characteristics that contribute to their development.

### Statistical analysis

The statistical study was conducted according to the database frozen in January 2019. The statistical analysis was performed using SPSS software, version 23. Kolmogorov Smirnov test was used to test the homogeneity of the variables. Data for patients were presented as means and standard deviation for variables normally distributed, while non-normally distributed data were reported as medians and interquartile ranges. Categorical variables were reported as numbers and percentages. The comparisons of AAU, psoriasis and IBD between the 2 groups of AS (with and without peripheral arthritis) and between disease durations (less than 15 years and more than 15 years) were examined using the Chi2 test or Fischer’s exact test. p values less than 0.05 were considered statistically significant. Baseline characteristics associated with the presence of AAU at baseline were identified with univariable followed by multivariable logistic regression analysis. Only characteristics often reported in the literature and those with a p-value <0.20 in the univariable analysis were entered in the multivariable analysis. Furthermore, the assumption that a variable could only be included in the model if there was a minimum of 10 patients with AAU at baseline was taken into account.

## RESULTS

170 patients, 34% of whom were women, were included in the present study. Their median age was 39 years. The mean disease duration was 12.2 years. HLA B27 was available in 50 patients and was positive in 33 patients. The total number of patients with a family history of SpA was 24 (15.3%). 67.5% of our patients had peripheral arthritis. <u>All</u> patients were treated with disease-modifying antirheumatic drugs (bDMARDs). Prevalence of EAMs was 13.5%, 4.7% and 11.2% for AAU, psoriasis and IBD respectively (**Table 1**).

**Table 1.** Demographic and clinical characteristics of AS patients in RBSMR.

**Characteristics**	**Value (N=170)**

Age (years) ^†^	39 [28–51]

Gender ^‡^	
Male	112 (66)
Female	58 (34)

Mean disease duration (years) (N=127) ^†^	12.2 ± 6.8

HLA B27 (N=50) ^‡^	
Positive	33 (66)
Negative	17 (34)

Family history of SpA (N=157) ^‡^	
Yes	24 (15.3)
No	133 (84.7)

Peripheral arthritis (N=166) ^‡^	
Present	112 (67.5)
Absent	54 (32.5)

**Previous bDMARD (N=38)** ^**‡**^	
Etanercept	10 (26.3)
Adalimumab	9 (23.7)
Infliximab	17 (44.7)
Golimumab	2 (5.3)

**Current bDMARD** ^**‡**^	
Etanercept	58 (34.1)
Adalimumab	51 (30)
Infliximab	42 (24.7)
Golimumab	17 (10)
Secukinumab	2 (1.2)

Extra-articular Manifestations ^‡^	
Acute anterior uveitis	23 (13.5)
Psoriasis	8 (4.7)
Inflammatory bowel disease	19 (11.2)

HLA B27: Human leukocyte antigen B27, SpA: spondyloarthritis, bDMARD: biological disease-modifying anti-rheumatic drug

† Median and interquartile range

‡ Number and percentage

† Means and ecart type

The prevalence of these EAMs was more pronounced after 15 years of AS progression, although the result was not significant (**Table 2**).

**Table 2.** Comparison of prevalence of acute anterior uveitis, psoriasis, and inflammatory bowel disease according to the disease duration of AS.

**Extra-articular Manifestations**	**Disease**	**duration**	**p**

	**Less than 15 years(N=88)**	**More than 15 years (N=39)**	

Acute anterior uveitis ^†^	6 (6.8)	8 (20.5)	0.7
Psoriasis ^†^	2 (2.3)	3 (7.7)	0.9
Inflammatory bowel disease ^†^	5 (5.7)	8 (20.5)	1

† Numbers and percentages

Patients with peripheral arthritis had higher prevalence of AAU (14.3% vs. 13%), psoriasis (6.2% vs. 1.8%) and IBD (11.6% vs. 11.1%) compared to patients without peripheral arthritis, even if these differences were not statistically significant (**Table 3**).

**Table 3.** Comparison of prevalence of acute anterior uveitis, psoriasis, and inflammatory bowel disease between AS with and without peripheral arthritis.

**Extra-articular Manifestations**	**AS without peripheralarthritis (N=54)**	**AS with peripheral arthritis (N=112)**	**p**
Acute anterior uveitis ^†^	7 (13)	16 (14.3)	0.69
Psoriasis ^†^	1 (1.8)	7 (6.2)	0.26
Inflammatory bowel disease ^†^	6 (11.1)	13 (11.6)	0.87

† Numbers and percentages

To assess the variables related to the presence of AAU, we used a multivariable analysis that included gender, disease duration, HLA B27 positive, family history of SpA, and peripheral arthritis. In the resulting model, family history of SpA increased the risk of AAU seven-fold (IC95%: 2.23–23.24). This association was statistically significant (p: 0.001), whereas there was no significant association with HLA B27 (**Table 4**).

**Table 4.** Factors associated with the presence of acute anterior uveitis: univariable and multivariable analysis.

	**Univariable**	**analysis**		**Multivariate**	**analysis**	
	**OR**	**IC 95%**	**p**	**OR**	**IC 95%**	**p**
**Gender (men)**	0.85	0.32–2.22	0.75			
**Disease duration**	0.72	0.22–2.30	0.58			
**HLA B27 positive**	3.57	0.68–18.71	0.13	0.51	0.09–2.84	0.44
**Presence of family history of SpA**	4.80	1.71–13.46	0.003	7.21	2.23–23.24	0.001
**Presence of peripheral arthritis**	0.82	0.31–2.15	0.70			

SpA: Spondyloarthritis

In our study, neither psoriasis was associated in binary logistic regression with peripheral arthritis and disease duration (p: 0.22 and 0.25 respectively), nor IBD was associated with HLA B27 positive and disease duration (p: 0.38 and 0.99 respectively).

## DISCUSSION

In Morocco, our study is the first one based on data collected from 10 departments of rheumatology in all Moroccan university medical centres that describe the prevalence of EAMs (AAU, psoriasis and IBD) in AS and analyse their risk factors. For patients with AS, EAMs occurs frequently, most notably AAU, IBD and psoriasis (7,8). As data showed, the AAU was the most common EAM in AS (13.5%). AAU is defined as a non-infectious acute inflammation of the anterior uveal tract and its adjacent structures. 20% to 30% of AS may develop AAU (8). Our result is compatible with all previous studies that reported a higher frequency of AAU in AS compared to psoriasis and IBD.^7,9^

In contrast, the reported prevalence of AAU (22–37%) was higher compared to our study (13.5%).^7,8,10^ It might be explained by a longer AS duration (15–17 years) compared to our study (12.2 years).

Although EAMs in our registry did not increase significantly with AS duration, Carmen Stolwijk et al. and Zeboulon et al. had demonstrated that prevalence of AAU increase with disease duration.^7,10^

Besides, some authors suggested that prevalence of IBD decrease after 10 years of AS diagnosis.^11^

Our data on peripheral arthritis in AS (67.5%) was higher than most published data (22.9%).^1^ A few clinical studies have reported similar higher incidence of peripheral arthritis (65.7%–70%).^12,13^

Comparison of the prevalence of these EAMs (AAU, psoriasis and IBD) between AS with and without peripheral arthritis, did not show a significant difference in contrast to some studies which have reported that patients with peripheral arthritis were more likely to have a higher prevalence of EAMs,^14,15^ even if the mechanism is not well-defined.^8^

Despite the high occurrence of AAU in AS, our understanding of risk factors for developing uveitis is still limited.^8,15^ An interesting observation from the current study is that a family history of SpA increased the risk of AAU seven-fold. However, surprisingly there was no association with HLA B27. Many studies have focused on the association between HLA B27 and uveitis and have proven a strong association; the presence of HLA B27 is associated with the development of 30% to 50% of AAU, and it is reported that HLA B27 positive individuals had a 3.8-fold greater chance to develop AAU than the HLA-B27 negative ones (8,16–18). This contradictory result might be explained by the low number of AS with HLA B27 typing in our registry. Prior studies have shown a clear association with AAU and disease duration,^5,15,19–20^ supported by systematic literature reviews,^7,10^ which had demonstrated that the prevalence of AAU increased with disease duration. Concerning variation of AAU in AS according to gender and peripheral arthritis, it was discussed in the literature with contradictory results.^8,14,21–22^

We found that the prevalence of psoriasis (4.7%) was less than reported in the literature (10%).^22,23^ Prior studies reported that psoriasis was more frequently associated with peripheral SpA, and that patients with psoriasis tend to exhibit more peripheral joint involvement.^4,24^ But this association cannot be supported by our findings.

IBD was not rare in our AS (11.2%) which is considerably higher than other studies.^7,9^ Studies on factors predictive of IBD development in AS are still rare.^25^ Some authors confirmed that HLA B27 is a predisposing factor for AS and patients with IBD seem to have a higher association with HLA B27 when compared to AS without IBD,^15^ whereas this was not the case for our data.

Up until now, studies had failed to prove the association between psoriasis and IBD with disease duration.^26^

Some limitations of the present study should be recognised. First, our RBSMR does not have HLA B27 typing of all patients. Moreover, in some AS, information was lacking on disease duration, family history of SpA and peripheral arthritis. Second, the number of EAMs are relatively small, which may reduce the power and strength of our conclusion. However, it is a large multicentric study enrolling patients from all the regions of Morocco.

## CONCLUSION

Awareness of EAMs is important in clinical practice because of their role in the diagnosis, treatment choices and for health-related quality of life. Our study, based on data of the RBSMR registry, summarized the prevalence of EAMs in AS; AAU was the most frequent EAM, and it was clearly associated with family history of spondyloarthritis. This data may be used as baseline comparators for further studies.

## Data Availability

The datasets are available from the RBSMR registry of the Moroccan Society of Rheumatology.
